# Dose-response relationship of blood flow restriction training on isometric muscle strength, maximum strength and lower limb extensor strength: A meta-analysis

**DOI:** 10.3389/fphys.2022.1046625

**Published:** 2022-12-15

**Authors:** Qun Yang, Xin Jia He, Ying Duan Li, Yong Zhi Zhang, Cong Shi Ding, Guo Xing Li, Jian Sun

**Affiliations:** Guangzhou Sport University, Guangzhou, China

**Keywords:** blood flow restriction, dose-response relationship, muscle strength, meta-analysis, lower limb muscle strength

## Abstract

**Objective:** To perform a meta-analysis on the efficacy and dose-response relationship of blood flow restriction training on muscle strength reported worldwide.

**Methods:** Thirty-four eligible articles with a total sample size of 549 participants were included in the meta-analysis. This study was performed using the method recommended by the Cochrane Handbook (https://training.cochrane.org/handbook), and the effect size was estimated using the standardized mean difference (SMD) and using RevMan 5.3 software (The Nordic Cochrane Centre, The Cochrane Collaboration, Copenhagen, 2014).

**Results:** The meta-analysis showed that blood flow restriction training increased the lower limb extensor muscle strength [SMD = 0.72, 95%; confidence interval (CI): 0.43 to 1.00, *p* < 0.01], knee extensor isokinetic torque SMD = 0.48 [95% CI: 0.24 to 0.73, *p* < 0.01], knee flexor isokinetic torque SMD = 0.39 [95% CI: 0.11 to 0.67, *p* < 0.01], and squat one-repetition maximum [SMD = 0.28, 95% CI: 0.01 to 0.55, *p* < 0.01]. There was no publication bias. Evaluation of dose-response relationship showed that the training load, mode, frequency, duration, and maximum cuff pressure affected the muscle function.

**Conclusion**: blood flow restriction training. 16 significantly improved lower limb muscle strength, and the optimal training conditions consisted of a weight load smaller or equal to 30% of one-repetition maximum, training duration longer than 4 weeks, frequency of more than 3 times/week, and maximum cuff pressure lower than 200 mmHg.

**Systematic Review Registration:** website, identifier registration number.

## 1 Introduction

Blood flow restriction training was introduced in 1983 as a novel training method in which the blood flow is restricted by applying pressure to the proximal muscle extremities with a specially designed constricting device, such as blood pressure cuff or tourniquet (Sheng et al., 2019). The blood flow restriction increases the mass of exercising muscles (Manini and Clark, 2009). It is also known as KAATSU training, with the advantages of low training risk and intensity but high frequency (Manini and Clark, 2009; Abe et al., 2012). In recent years, bloo^1^d flow restriction training has been widely used in competitive sports, public fitness, and rehabilitation. Studies have confirmed the beneficial effects of blood flow restriction training on athletes in track and field, soccer, rugby, tennis, handball, and basketball by improving the maximum strength and isokinetic torque strength of the upper and lower limb muscles (Kubota et al., 2008; Kubota et al., 2011; Xu and Wang, 2013; Pope et al., 2015; Tennent et al., 2017; Li et al., 2019; Wang et al., 2019). A meta-analysis by Wu et al. of 14 studies showed that blood flow restriction training increased the standard extensor strength of lower limbs and the cross-sectional area of quadriceps (Wu et al., 2019). Niu et al. conducted a meta-analysis of 47 studies and concluded that blood flow restriction training significantly increased muscles’ mass and maximum strength (Niu et al., 2020). Loenneke et al. found in a meta-analysis that 15%–30% one-repetition maximum (1RM)/maximal voluntary contraction (MVC) had a better muscle training effect (Abe et al., 2012). Similarly, Slysz et al. confirmed the effectiveness of blood flow restriction training on muscle hypertrophy and strength, with analysis of 47 studies (Slysz and Burr, 2016). Although the effects of blood flow restriction training on the body have been confirmed, there is still a lack of evidence-based on the “dose-response” relationship of blood flow restriction training (Manini and Clark, 2009). Duo to the above, this study is the first to systematically evaluate the intervention intensity, cycle, frequency, and pressure peak of blood flow restriction training, and conduct a four-item meta-analysis study on the effect of blood flow restriction training on lower limb muscle strength. In this study, a dose-effect relationship will be observed between blood flow restriction training and lower extremity muscle strength in order to evaluate the training effect more quantitatively (Aune et al., 2016) and to provide a more objective and reliable blood flow restriction training plan, in order to produce scientific and reliable results—quantitative guidance.

## 2 Methods

### 2.1 Search strategy and study selection

This meta-analysis was performed according to the Preferred Reporting Items for Meta-Analyses (PRISMA) guidelines (Cumpston et al., 2019). A computer-assisted literature search was performed in China National Knowledge Infrastructure, Wan-Fang Med Online, Baidu Scholar, Web of Science, PubMed, Elsevier, Embase, and EBSCOhost. The keywords were “pressurized training”, “blood flow restriction training”, “blood blockage; training”, and “KAATSU training”. The English keywords were “KAATSU training” [Title/Abstract] OR “blood flow restriction training” [Title/Abstract] OR “BFRT” [Title/Abstract] OR “blood flow restriction exercise” [Title/Abstract] OR “occlusion training” [Title/Abstract] “Restricted Leg Blood Flow” [Title/Abstract]. We searched articles published from inception to October 2022. All retrieved articles in Chinese and English were imported into EndNote (version X9, Clarivate Analytics) for deduplication. Two independent reviewers (HJX and ZZY) screened articles against the inclusion and exclusion criteria, and discrepancies were resolved by discussion and consensus with a third reviewer (SJ). The following data were extracted: 1) citation information of included studies, such as title, first author, journal, and time of publication; 2) baseline characteristics of participants, including a sample size of each group, and the age and sex of participants; 3) details of interventions such as duration, frequency, load, maximal cuff peak; 4) key elements of bias risk assessment; 5) outcome indicators and measures.

### 2.2 Inclusion criteria

According to the PICOS (Liu et al., 2017) principle (patient/population, intervention, comparison/control, outcome and study design), trials were eligible for inclusion if they met the criteria as follows: 1) participants were defined as healthy individuals; 2) the **t**ypes of studies include randomized controlled trials (RCTs) or self-controlled trials, regardless of blinding or allocation concealment, and the control groups were daily activities or regular physical activities without interventions. 3) the outcomes measures of the trial included functional indicators include knee flexor (extensor) isokinetic torque, maximum squat strength, and lower limb extensor strength. 4) the selected articles were peer-reviewed publications written in English.

### 2.3 Exclusion criteria

Trials were excluded when they met any of the following exclusion criteria: 1) Trials without randomized, self-controlled, or randomized crossover design; 2) lack of appropriate outcome indicators; 3) conference abstracts and overviews; 4) articles with inappropriate subjects (animals).

### 2.4 Quality assessment

The quality of articles as indicated by the risk of bias in RCT was assessed using the method recommended by the Cochrane Handbook (https://training.cochrane.org/handbook). The articles were ranked at high, low, or unclear risk of bias. The low, high, and unclear risk of bias is represented by three colors in the evaluation plot, green for low, yellow for uncertain, and red for high.

### 2.5 Statistical analysis

RevMan, Computer program, version 5.3 (The Nordic Cochrane Centre, The Cochrane Collaboration, Copenhagen, 2014) and using STATA software version 17 (STATA Corp, College Station, TX, USA) for data analysis. Q test was used to assess the heterogeneity of data in articles (*α* = 0.1). The effect size was calculated using different models based on the heterogeneity between studies. *I* ^2^ ≤ 50% (*p* ≥ 0.1) was regarded as minimal heterogeneity between studies, and a fixed-effects model was adopted. A random-effects model was used otherwise. The effect size was expressed as standardized mean difference (SMD), 95% CI, and subgroup analysis for moderators, with effect sizes of 0.2, 0.5, and 0.8 defined as small, medium, and medium-large, respectively.

## 3 Results

### 3.1 Literature review

**FIGURE 1:**
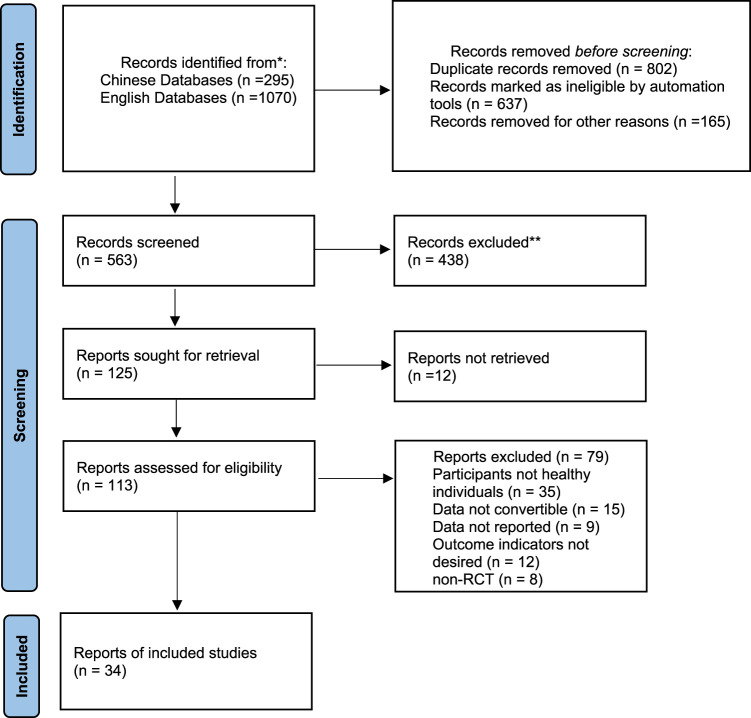
the flow diagram of the search procedure.

A total of 1,070 English articles and 295 Chinese articles were retrieved, and 29 English articles (Abe et al., 2005; Beekley et al., 2005; Yasuda et al., 2005; Fujita et al., 2008; Madarame et al., 2008; Abe et al., 2010a; Abe et al., 2010b; Park et al., 2010; Sumide et al., 2010; Madarame et al., 2011; Tomohiro et al., 2011; Yasuda et al., 2011; Godawa et al., 2012; Keramidas et al., 2012; Martín-Hernández et al., 2012; Tetsuo et al., 2012; Luebbers et al., 2014; Yasuda et al., 2014; Barcelos et al., 2015; Fahs et al., 2015; Lixandrão et al., 2015; Vechin et al., 2015; Yasuda et al., 2015; Kim et al., 2016; Luebbers et al., 2017; Slysz and Burr, 2017; Bjørnsen et al., 2019; Harper et al., 2019; Schwiete et al., 2021; Teixeira et al., 2021) and 5Chinese articles (Liu et al., 2018; Lu et al., 2020; Zhang et al., 2020; Li et al., 2022; Tang and Qu, 2022) were included in the meta-analysis. The flow diagram of the study selection is shown in Figure 1.

### 3.2 Basic information of the included studies

A total of 34 articles with 49 studies (with some articles containing multiple studies) were included for 145 meta-analysis, with 549 participants in intervention groups and 549 participants in control groups. Participants were of mixed gender, age between 18 and 78 years and were males (70%) and were females (30%). RCT were used in all studies with blood flow restriction training as the intervention. The training load weight (based on one-repetition maximum) is divided into three categories: a low load (20%–50% 1RM), a medium load (50%–80% 1RM), and a high load (80%–100% 1RM). The training could be short-term training (1–2 weeks) or chronic, and the duration, frequency, and cuff pressure are shown in Table 1.

**TABLE 1:** Basic information of included articles.

Authors	Participants	Age (y)	Sample size (C/T)	Load	Frequency	Duration	Cuff pressure (mmHg)	Outcome indicators
Li et al	Athletes	22.29 ± 3.27	8/8	30% 1RM	3 times/w	4 w	200	II, III, IV
Wang et al	Athletes	24.2 ± 4.2	9/9	30% 1RM	3 times/w	8 w	200–220	II, III
Lu et al	Healthy individuals	22.4 ± 1.94	9/9	20% 1RM	3 times/w	12 w	120–180	II
Abe et al. b	Athletes	*	6/9	20% 1RM	2 times/d	8 d	160–240	IV
Abe et al. e	Healthy individuals	60–78	8/11	45% HRmax	5 times/w	6 w	160–230	I, II, III
Abe et al. f	Healthy individuals	23.0 ± 1.7	10/9	45% HRmax	3 times/w	8 w	160–230	II, III
Beekley et al	Healthy individuals	21.3 ± 2.8	9/9	80% 1RM	2 times/d	3 w	160–230	IV
Fahs et al	Healthy individuals	55 ± 7	9/9	30% above 1RM	2 times/w	6 w	120–300	IV
Fujita et al	Healthy individuals	22.3 ± 2.9	8/8	20% 1RM	2 times/d	6 d	200	I
Godawa et al	Athletes	21 ± 2.45	9/9	80% 1RM	2 times/w	10 w	*	IV
Keramidas et al	Healthy individuals	22.7 ± 4.7	10/10	50%–80%	3 times/w	6 w	90	II, III
VO2max								
Kim et al	Healthy individuals	22.4 ± 3	11/11	30% 1RM	3 times/w	6 w	120–160	I
Laurentino et al	Healthy individuals	22.42 ± 3.41	8/8	60%–80% 1RM	2 times/w	8 w	130	I
Letieri et al	Healthy individuals	68.8 ± 5.09	13/13	20%–30% 1RM	3 times/w	6 w	80–130	I
Lixandr et al	Healthy individuals	29.2 ± 9.9	9/11	20%–40% 1RM	1 time/w	12 w	110	IV
Luebbers et al. a	Athletes	20.3 ± 1.1	14/17	20% 1RM	2 times/w	7 w	200	I
Luebbers et al. b	Athletes	15.8 ± 1.2	8/8	60%–85% 1RM	2 times/w	6 w	200	I
Madarame et al	Healthy individuals	21.6 ± 2.4	7/8	30% 1RM	2 times/w	10 w	160–240	I
Marítn-Hernández et al	Healthy individuals	20.3 ± 1.1	8/10	20% 1RM	1 time/w	5 w	110	I, II, III
Park et al	Athletes	20.1 ± 1.2	5/7	40% 1RM	2 times/w	4 w	160–220	II, III
Slysz et al	Healthy individuals	18–45	10/10	Unknown	4 times/w	6 w	220	IV
Sumide et al	Healthy individuals	22.1 ± 1.8	5/6	20% 1RM	3 times/w	8 w	150	II
Vechin et al	Healthy individuals	59–71	7/8	50% 1RM	2 times/w	12 w	7/10	IV
Yasuda et al	Healthy individuals	22–28	10/10	75% 1RM	3 times/w	6 w	100–160	IV
Yasuda et al. a	Healthy individuals	22–32	10/10	30% 1RM	2–3	6 w	100–130	IV
								times/w
Yamanaka et al	Athletes	19.2 ± 1.8	16/16	60% 1RM	1 time/w	4 w	50–100	I, IV
Yasuda et al. b	Healthy individuals	71.3 ± 7.1	10/10	20%–30% 1RM	3 times/w	12 w	120–270	I
Yasuda et al. c	Healthy individuals	61–78	8/8	80% 1RM	2 times/w	24 w	100–160	I
Barcelos et al	Healthy individuals	18–30	10/10	20% 1RM	3 times/w	8 w	200	II
Madarame et al. a	Healthy individuals	19.9 ± 1.6	7/9	30% 1RM	2 times/w	10 w	200–250	I, II, III
Tang et al	male road cyclists	22.73 ± 1.85	6/6	40% 1RM	2 times/w	4 w	40–200	II, III
Li et al	male tennis players	19.7 ± 3.2	10/10	30% 1RM	2 times/w	6 w	200	IV
Teixeira et al	Healthy individuals	26.0 ± 4 0.0	10/10	30% 1RM	2 times/w	3 w	102	II
Schwiete et al	trained participants	22.8 ± 1.8	10/9	50% 1RM	2 times/w	1 w	180	IV

Note: I, standard lower limb extensor muscle strength; II, knee extensor isokinetic torque; knee flexor isokinetic torque; IV, squat.

III, 1RM.* represents no data.

### 3.3 Bias risk assessment

**FIGURE 2:**
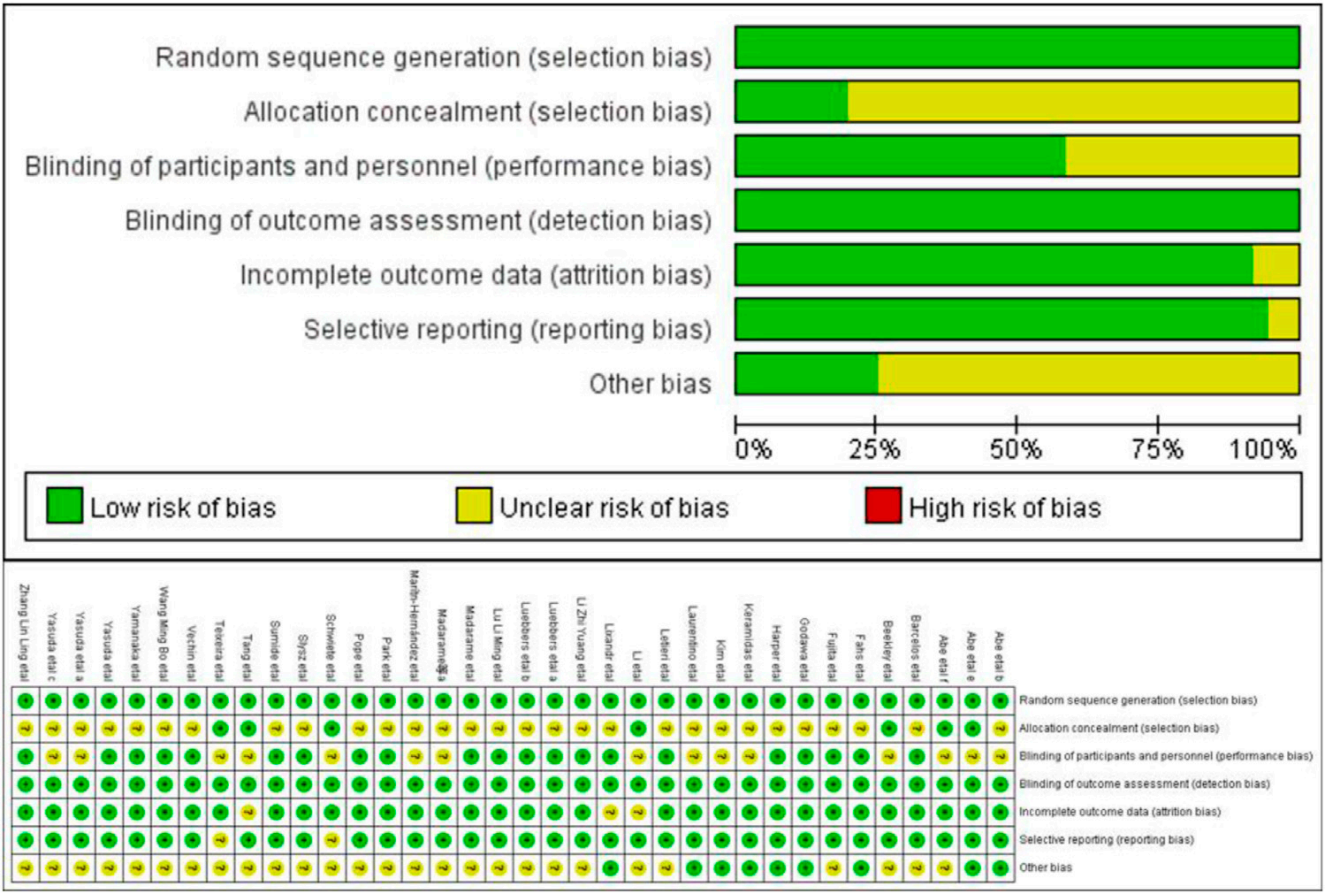
The bias of the included studies. Note: “+” represents low risk, “−” high risk, and “?” unclear.

The quality of 34 articles with RCT was evaluated with Cochrane risk assessment tool. Three articles mentioned allocation concealment, and the risk of bias was low. The remaining articles mentioned randomization through detailed methods, and allocation concealment was absent, therefore the risk of bias was unclear. In the 23 articles using blinding the risk of bias was low. All publications reported the loss of follow-up and were of high quality (Figure 2).

### 3.4 Overall effect of blood flow restriction training on lower limb muscle function

#### 3.4.1 Muscular strength indicators

**FIGURE 3:**
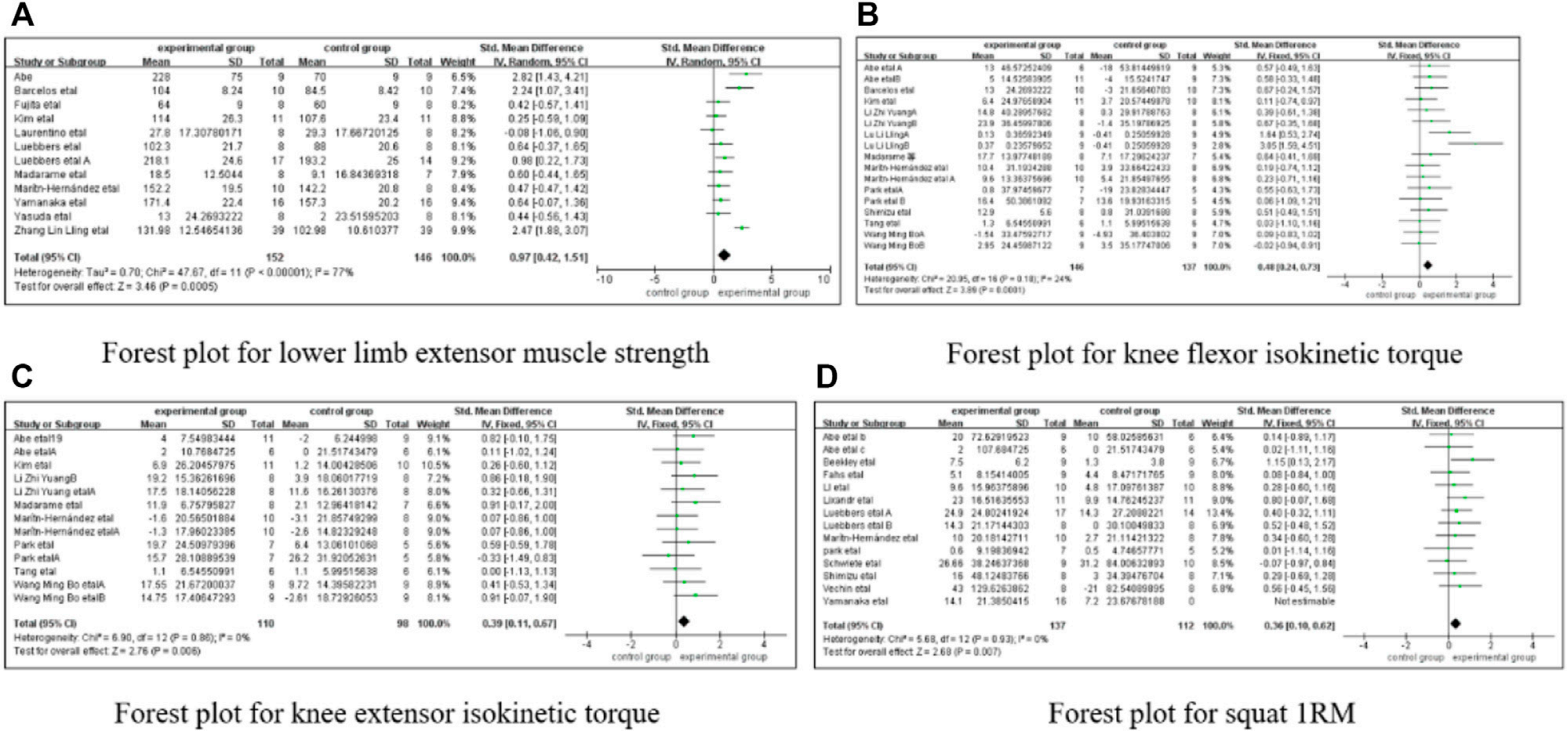
The forest plot of four outcome indicators. **(A)** Forest plot for lower limb extensor muscle strength, **(B)** Forest plot for knee flexor isokinetic torque, **(C)** Forest plot for knee extensor isokinetic torque, **(D)** Forest plot for squat IRM.

The functional indicators described in the 34 articles added to the present analysis were categorized into knee extensor isokinetic torque, knee flexor isokinetic torque, the lower limb extensor muscle strength, and squat one-repetition maximum (1RM). A total of 11 studies with 220 participants were included for the lower limb extensor muscle strength, with 11 individual effect sizes, an overall effect size of SMD = 0.72 [95% CI: 0.43 to 1.00, *p* < 0.01], and heterogeneity (*I* ^2^ = 51%, *p* = 0.02). Seventeen studies with 283 participants were included for knee extensor isokinetic torque, with 17 individual effect sizes, an overall effect size of SMD = 0.48 [95% CI: 0.24 to 0.73, *p* < 0.01], and no heterogeneity (*I* ^2^ = 24%, *p* = 0.18). A total of 13 studies with 208 participants were included for knee flexor isokinetic torque, with 13 individual effect sizes, an overall effect size of SMD = 0.39 [95% CI: 0.11 to 0.67, *p* < 0.01], and no heterogeneity (*I* ^2^ = 0%, *p* = 0.86). Fourteen studies with 249 participants were included for squat 1RM, with 14 individual effect sizes, an overall effect size of SMD = 0.36 [95% CI:0.10 to 0.62, *p* < 0.01], and no heterogeneity (*I* ^2^ = 0%, *p* = 0.93). This above are shown in Figure 3.

#### 3.4.2 Dose-response relationship

Based on the included articles, the dose-response relationship was evaluated in the training load, training duration, training frequency, and maximum cuff pressure.

##### 3.4.2.1 Effect of blood flow restriction training on knee extensor isokinetic torque

1) Training load. A total of 18 studies with 290 participants were included in this group. A training load of ≤30% 1RM showed a higher effect size of d = 0.583 [SMD = 0.583, 95% CI: 0.279 to 0.852, *p* < 0.01]. A training load of >30% 1RM showed an effect size of d = 0.564 [SMD = 0.564, 95% CI: 0.215 to 0.869, *p* < 0.01]. Both conditions achieved moderate training effects. The Cohen effect sizes <0.20 were considered small, 0.20 < d < 0.80 moderate, and ≥0.8 large. The above data indicate that ≤30% 1RM achieved better training effect.
2) Training duration. A total of 18 studies with 290 participants were included in this group. A training duration of >4 weeks showed a higher effect size of d = 0.636 [SMD = 0.636, 95% CI: 0.309 to 0.963, *p* < 0.01]. A training duration of ≤4 weeks showed an effect size of d = 0.589 [SMD = 0.589, 95% CI: 0.032 to 0.782, *p* < 0.01]. Both conditions achieved moderate training effects. The above data suggest that >4 weeks achieved better training effect.
3) Training frequency. A total of 18 studies with 290 participants were included in this group. A training frequency of ≥3 times/week showed a higher effect size of d = 0.622 [SMD = 0.622, 95% CI:0.325 to 0.919, *p* < 0.01]. A training frequency of 1–2 times/week showed an effect size of d = 0.423 [SMD = 0.423, 95% CI: −0.062 to 0.672, *p* > 0.05]. The above data suggest that ≥3 times/week achieved better training effect.
4) Maximum cuff pressure. A total of 18 studies with 290 participants were included in this group. A 201 maximum cuff pressure of ≤200 mmHg showed a higher effect size of d = 0.668 [SMD = 0.668, 95% CI: 0.353 to 0.983, *p* < 0.01], whereas a maximum cuff pressure of >200 mmHg showed an effect size of d = 0.306 [SMD = 0.306, 95% CI: −0.110 to 0.721, *p* < 0.05]. The above data indicate that a maximum cuff pressure of ≤200 mmHg achieved better training effect. This above are shown in Table 2.

**TABLE 2:** Subgroup analysis of blood flow restriction training effect on knee extensor isokinetic torque.

Factor	Subgroup	Number of articles	Knee flexor isokinetic torque	Heterogeneity		
			SMD (95% CI)	*p*	*I* ^2^ (%)	P
Training load	>30% 1RM	5	0.564 (0.215, 0.869)	0	0	0.828
	≤30% 1RM	15	0.583 (0.279, 0.852)	0	50	0.024
Training duration	>4 weeks	10	0.636 (0.309, 0.963)	0	61.2	0.008
	≤4 weeks	10	0.589 (0.032, 0.782)	0.036	0	0.966
Training frequency	1–2 times/week	8	0.423 (−0.062, 0.672)	0.946	0	0.183
	≥3 times/week	11	0.622 (0.325, 0.919)	0	51.9	0.022
Maximum cuff pressure	>200	6	0.306 (−0.110, 0.721)	0.151	0	0.882
	≤200	14	0.668 (0.353,0.983)	0	52.8	0.025

##### 3.4.2.2 Effect of blood flow restriction training on knee flexor isokinetic torque

1) Training load. A total of 13 studies with 208 participants were included in this group, and training load of ≤30% 1RM showed a higher effect size of d = 0.442 [SMD = 0.442, 95% CI: 0.154 to 0.730, *p* < 0.01]. Training load of da>30% 1RM showed effect size of d = 0.001 [SMD = 0.001, 95% CI: 0.389 to 0.823, *p* > 0.05]. The above data indicate that ≤30% 1RM achieved a good training effect.
2) Training duration. A total of 13 studies with 208 participants were included in this group. A training duration of >4 weeks showed a higher effect size of d = 0.606 [SMD = 0.606, 95% CI: 0.389 to 0.823, *p* < 0.01]. A training duration of ≤4 weeks showed an effect size of d = 0.583 [SMD = 0.583, 95% CI: 0.053 to 1.018, *p* < 0.01]. Both conditions achieved moderate training effects. The above data suggest that a training duration >4 weeks achieved better training effect.
3) Training frequency. A total of 13 studies with 208 participants were included in this group. A training frequency of ≥3 times/week showed a higher effect size of d = 0.463 [SMD = 0.463, 95% CI: 0.140 to 0.787, *p* < 0.01]. A training frequency of 1–2 times/week showed an effect size of d = 0.289 [SMD = 0.289, 95% CI: −0.205 to 0.672, *p* > 0.05]. The above data suggest that a frequency ≥3 times/week achieved better training effect.
4) Maximum cuff pressure. A total of 13 studies with 208 participants were included in this group. A maximum cuff pressure of ≤200 mmHg showed a higher effect size of d = 0.683 [SMD = 0.683, 95% CI: 0.018 to 0.799, *p* < 0.01]. A maximum cuff pressure of >200 mmHg showed an effect size of d = 0.559 [SMD = 0.559, 95% CI: 0.165 to 0.953, *p* < 0.01]. Both conditions achieved moderate training effects. The above data indicate that a maximum cuff pressure ≤200 mmHg achieved better training effect (Table 3).

**TABLE 3:** Subgroup analysis of blood flow restriction training effect on knee flexor isokinetic torque.

Factor	Subgroup	Number of articles	Knee flexor isokinetic torque		Heterogeneity	
			SMD (95% CI)	*p*	*I* ^2^ (%)	*p*
Training load	≤30% 1RM	14	0.442 (0.154, 0.730)	0.003	0	0.781
Training duration	>4 weeks	7	0.632 (0.133, 0.846)	0.007	0	0.670
	≤4 weeks	5	0.522 (0.033, 1.011)	0.036	0	0.926
Training frequency	1–2 times/week	4	0.277 (−0.226, 0.780)	0.28	0	0.569
	>3 times/week	8	0.463 (0.140, 0.787)	0.005	0	0.672
Maximum cuff pressure	>200	7	0.559 (0.165, 0.953)	0.005	0	0.588
	≤200	5	0.669 (0.003, 0.789)	0.048	0	0.692

##### 3.4.2.3 Effect of blood flow restriction training on lower limb extensor muscle strength

1) Training load. A total of 11 studies with 220 participants were included in this group. A training load of ≤30% 1RM showed a higher effect size of d = 0.880 [SMD = 0.880, 95% CI: 0.494 to 1.2660, *p* < 0.01]. A training load of >30% 1RM showed an effect size of d = 0.548 [SMD = 0.548, 95% CI: 0.098 to 0.999, *p* < 0.05]. Both conditions achieved moderate training effects. The above data indicate that ≤30% 1RM achieved better training effect.
2) Training duration. A total of 11 studies with 220 participants were included in this group. A training duration of >4 weeks showed a higher effect size of d = 1.109 [SMD = 1.109, 95% CI: 0.818 to 1.400, *p* < 0.01]. A training duration of ≤4 showed an effect size of d = 0.943 [SMD = 0.943, 95% CI: 0.409 to 1.477, *p* < 0.01]. Both conditions achieved moderate training effects. The above data suggest that a training duration >4 weeks achieved better training effect.
3) Training frequency. A total of 11 studies with 220 participants were included in this group. A training frequency of ≥3 times/week showed a higher effect size of d = 0.653 [SMD = 0.653, 95% CI: 0.363 to 0.941, *p* < 0.01]. A training frequency of 1–2 times/week showed an effect size of d = 2.958 [SMD = 2.958, 95% CI: 1.578 to 4.338, *p* > 0.05], and the risk of publication bias was high because there was only one trial. The above data suggest that a training frequency ≥ three times/week achieved a better effect.
4) Maximum cuff pressure. A total of 11 studies with 220 participants were included in this group. A maximum cuff pressure of ≤200 mmHg showed a higher effect size of d = 0.653 [SMD = 0.653, 95% CI: 0.352 to 0.954, *p* < 0.01]. A maximum cuff pressure of >200 mmHg showed an effect size of d = 0.566 [SMD = 0.566, 95% CI: 0.337 to 0.796, *p* < 0.01]. Both conditions achieved moderate training effects. The above data indicate that a maximum cuff pressure ≤200 mmHg achieved better training effects (Table 4).

**TABLE 4:** Subgroup analysis of blood flow restriction training effect on lower limb extensor muscle strength.

Factor	Subgroup	Number of articles	Lower limb extensor muscle strength		Heterogeneity	
			SMD95%CI	p	I2%	p
	>30%1RM	4	0.548 (0.098, 0.999)	0.017	1.9	0.383
Training load						
	≤30%1RM	8	0.880 (0.766, 1.266)	0	86.4	0
	>4 weeks	10	1.109 (0.818, 1.400)	0	79.8	0
Training duration						
	≤4 weeks	7	0.943 (0.409, 1.477)	0.001	76.9	0.005
	1–2 times/w	6	2.958 (1.578, 4.338)	0.005	81	0.004
Training frequency						
	≥3 times/w	11	0.653 (0.363, 0.940)	0	80	0.001
	>200 mmHg	2	0.566 (0.337, 0.769)	0	85.5	0
Maximum cuff pressure						
	≤200 mmHg	13	0.653 (0.352, 0.954)	0	35.4	0.135

##### 3.4.2.4 Effect of blood flow restriction training on squat 1-RM

1) Training load. A total of 18 studies with 448 participants were included in this group. A training load of >30% 1RM showed a higher effect size of d = 0.639 [SMD = 0.639, 95% CI: 0.389 to 0.889, *p* < 0.01]. A training load of ≤30% 1RM showed an effect size of d = 0.325 [SMD = 0.325, 95% CI: 0.110 to 0.638, *p* < 0.01]. Both conditions achieved moderate training effects. The above data indicate that ≤30% 1RM achieved better training effect.
2) Training duration. A total of 18 studies with 448 were included in this group. A training duration of >4 weeks showed a higher effect size of d = 0.606 [SMD = 0.606, 95% CI: 0.389 to 0.823, *p* < 0.01]. A training duration of ≤4 weeks showed an effect size of d = 0.413 [SMD = 0.413, 95% CI: 0.047 to 0.873 *p* < 0.05]. Both conditions achieved moderate training effects. The above data indicate that >4 weeks achieved better training effect.
3) Training frequency. A total of 17 studies with 455 participants were included in this group. A training frequency of ≥3 times/week showed a higher effect size of d = 0.550 [SMD = 0.550, 95% CI: 0.349 to 0.752, *p* < 0.01], whereas a training frequency of 1–2 times/week showed an effect size of d = 0.629 [SMD = 0.429, 95% CI: 0.262to 0.790, *p* < 0.01]. Both conditions achieved moderate training effects. The above data indicate that a training frequency of 1–2 times/week achieved better training effect.
4) Maximum cuff pressure. A total of 18 studies with 455 participants were included in this group. A maximum cuff pressure of ≤200 mmHg showed a higher effect size of d = 0.653 [SMD = 0.653, 95% CI: 0.195 to 0.867, *p* < 0.01]. A maximum cuff pressure of >200 mmHg showed an effect size of d = 0.566 [SMD = 0.566, 95% CI: 0.337 to 0.796, *p* = 0.05]. Both conditions achieved moderate training effects. The above data indicate that a maximum cuff pressure ≤200 mmHg achieved better training effect. This above are shown in Table 5.

**TABLE 5:** Subgroup analysis of blood flow restriction effect on Squat1-RM.

Factor	Subgroup	Number of articles	Squat1-RM	Heterogeneity			
			SMD 95% CI	P	*12* (%)	P
Training load	>30%1RM	19	0.325 (0.110, 0,638)	0.003	0	0.677
	≤30%1RM	8	0.639 (0.389, 0.889)	0.002	0	0.879
Training duration	>4 weeks	8	0.606 (0.389,0.823)	0.005	0	0.965
	≤4 weeks	4	0.413 (−0.047, 0.873)	0.049	5	0.368
Training frequency	1-2times/w	2	0.629 (0.262, 0.790)	0.005	0	0.897
	≥3times/w	10	0.550 (0.349, 0.752)	0.002	0	0.861
Maximum cuff pressure	>200 mmHg	6	0.566 (0.337, 0.796)	0.05	0	0.545
	<200 mmHg	6	0.653 (0.195, 0.867)	0.011	0	0.956

### 3.5 Publication bias and sensitivity analysis

#### 3.5.1 Publication bias

**FIGURE 4:**
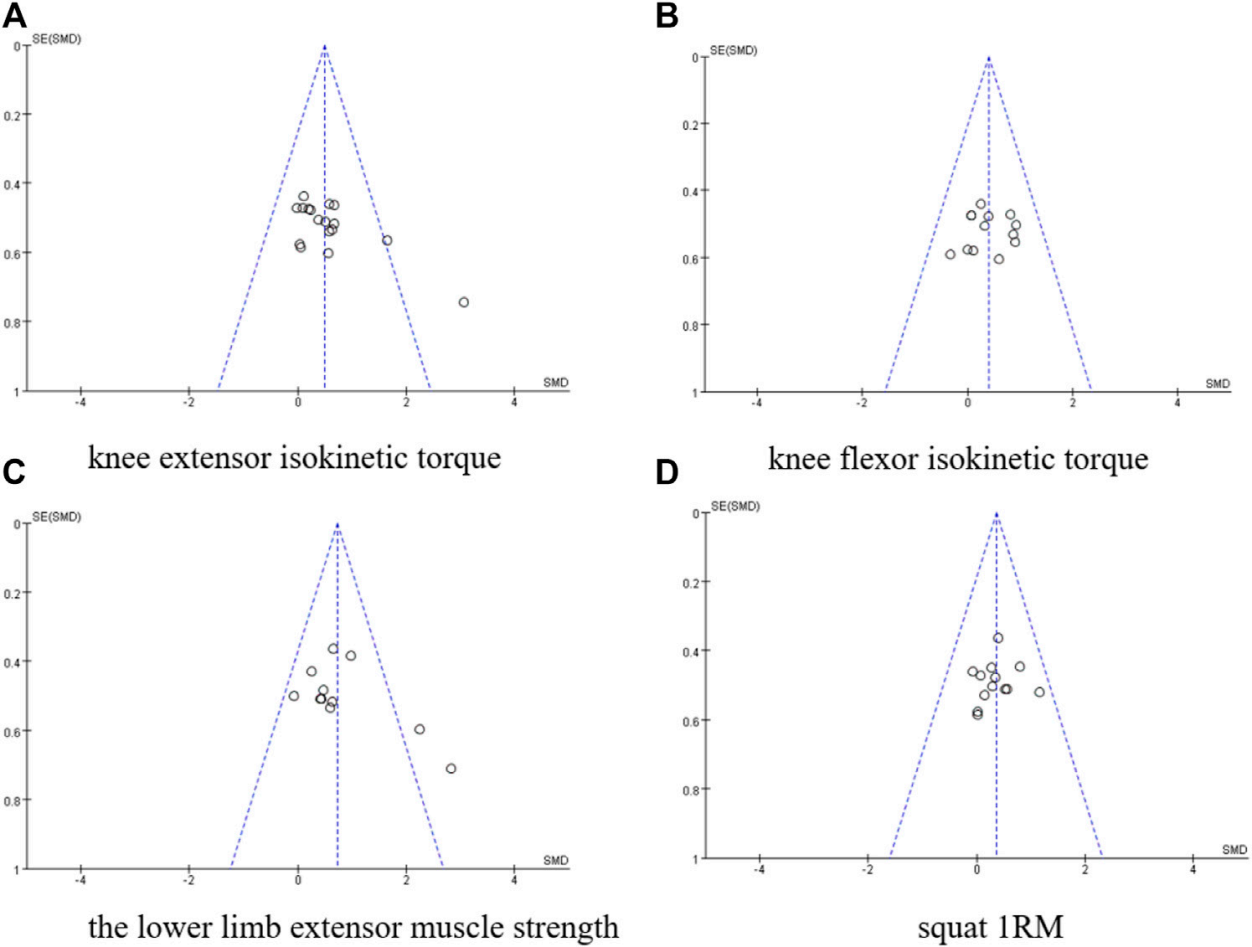
Publication bias plot. **(A)** knee extensor isokinetic torque, **(B)** knee flexor isokinetic torque, **(C)** The lower limb extensor muscle strength, **(D)** Squat IRM.

Begg’s funnel plot was used to assess the publication bias of knee extensor’s, knee flexor isokinetic torque, lower limb extensor muscle strength, and squat 1RM. The symmetrical funnel plots suggested no publication bias for the four indicators. This above are shown in Figure 4.

#### 3.5.2 Sensitivity analysis

**FIGURE 5:**
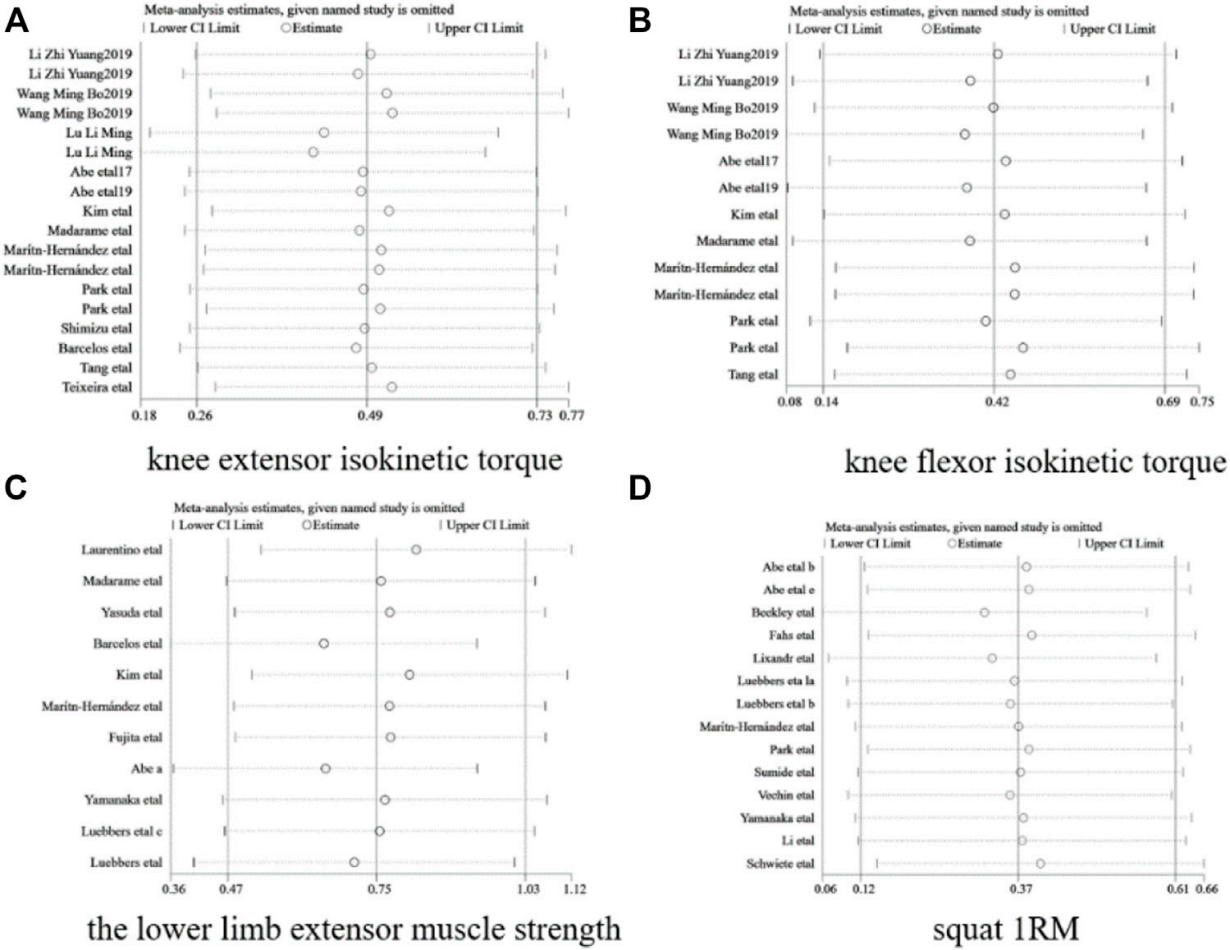
Sensitivity analysis. **(A)** knee extensor isokinetic torque, **(B)** knee flexor isokinetic torque, **(C)** The lower limb extensor muscle strength, **(D)** Squat IRM.

The sensitivity analysis is based on knee extensor’s, knee extensor’s, torque, lower limb extensor muscle strength, and squat 1RM. Sensitivity analysis was performed using the leave-one-out method, in which dubious literature was excluded one by one to test for the presence of literature that significantly influenced the conclusion. The results are shown in Figure 5, which shows that after excluding any study, the degree of bias of the point estimates of the new combined effect sizes obtained by subjecting the remaining studies to a new Meta-analysis was not significant, suggesting good robustness of the conclusion and that no single study significantly influenced the conclusion, so all included literature was retained. This above are shown in Figure 5.

## 4 Discussion

Blood flow restriction training had a positive effect on participant’s lower limb muscle function, and all studies showed moderate effect sizes for function improvement. Comparative analysis showed that Blood flow restriction training increased the knee extensor isokinetic torque, knee flexor isokinetic torque, lower limb extensor muscle strength, and squat 1RM. In particular, the improvement of lower limb extensor muscle strength was high and with low heterogeneity. Load measured by percentages of 1RM and MVC is an important factor influencing the training effect, and 20%–30% 1RM has been recommended for optimal training performance (Manini and Clark, 2009), which was confirmed in the present study. Abe et al. used 20% 1RM for a 2-week blood flow restriction training of healthy male participants (Abe et al., 2012). They found significant increases in the maximum squat strength (16.8%) and leg curl strength (22.6%) for 2 weeks (Zhang et al., 2020), similar to the training effect in another study using 30% 1RM over 10 weeks (Luebbers et al., 2017). Although the training effect increases with training load, an excessive load may not lead to beneficial results. Jessee et al. compared 50% 1RM with 30% 1RM and found little difference in muscle hypertrophy (Madarame et al., 2011; Jessee et al., 2018). Moreover, studies showed that blood flow restriction training did not increase muscle activation at high load (70% 1RM) (Takarada et al., 2002) and decreased muscle activation at > 70% 1RM (Dankel et al., 2017). In addition, blood flow restriction training at 80% 1RM showed no significant improvement in muscle hypertrophy and strength (Luebbers et al., 2014; Winchester et al., 2020). As muscle resistance increases, muscle contraction has internal restrictions on blood flow, and external cuff pressure may fail to produce a positive effect. In addition, excessive blood flow restriction causes severe fatigue (Teixeira et al., 2018). Based on previous findings (Niu et al., 2020), the effect may be optimal at training loads not exceeding 30% 1RM.

### 4.1 Training duration

The studies of the training duration showed that the effect size increased with the number of weeks of training and reached a maximum at ≥ 4 weeks. Blood flow restriction training increases not only muscle hypertrophy and strength but also the adaptation of muscles to physiological load. Therefore, the training should be performed in a low-load, high-frequency, and incremental manner.

### 4.2 Training frequency

The frequency of blood flow restriction training ranged from 2 to 5 times/week (5 days per week) (Yasuda et al., 2005; Liu et al., 2018). Fujita et al. found that the maximum strength of the knee extensor was increased after 6 days (twice a day) of training at 20% 1RM (Fujita et al., 2008). In contrast, blood flow restriction training to subjective exhaustion was reported to reduce training frequency (Winchester et al., 2020). Research has shown that although acute blood flow restriction training enhanced muscle activation, low-intensity blood flow restriction training was less effective than high-intensity resistance training in improving muscle strength; however, the effect of blood flow restriction training gradually appeared with the increase of training frequency (Fitschen et al., 2015). The present study showed that the performance was optimal at > 3 times/week.

### 4.3 Maximum cuff pressure

Cuff pressure is an important factor affecting blood flow restriction training and the blood flow restriction in extremities, and thus a fundamental condition of this training method. Cuff pressure causes difficulty in convective oxygen transport, leading to the accumulation of metabolites such as lactate and increased secretion of the growth hormone (Takano et al., 2005; Fatela et al., 2018), thus achieving similar effects as high-intensity exercises. The peak-value effect size varied in the range of cuff pressure (80–200 mmHg) applied in the analyzed studies, and the data suggest that a maximum cuff pressure ≤ of 200 mmHg had the best effect on increasing muscle function.

## 5 Practical limitations

Based on the above analyses, the following aspects should be noted in future studies. 1) A meta-analysis should be based on a comprehensive collection of previous studies, and the unavailability of unpublished articles and unreported negative results should always be addressed. 2) In this study, we only considered the effects of blood flow restriction training on muscle strength indicators, and future studies could include muscle function, safety, and gender differences. 3) The dose-response relationship for the elderly and rehabilitation populations needs to be further explored. 4) The participants in the included studies could have very different training status. 5) The age range was quite varied (18–78). Aging effects should be further concerned.

## 6 Conclusion

Blood flow restriction training significantly improved lower limb muscle strength. The optimal blood f1ow restriction training conditions observed in this meta-analysis were a training load of ≤30% 1RM, training duration of >4 weeks, training frequency of >3 times/week, and a maximum cuff pressure of ≤200 mmHg.

## Data Availability

The original contributions presented in the study are included in the article/supplementary material, further inquiries can be directed to the corresponding author.
